# Transcriptional signature of human pro-inflammatory T_H_17 cells identifies reduced *IL10* gene expression in multiple sclerosis

**DOI:** 10.1038/s41467-017-01571-8

**Published:** 2017-11-17

**Authors:** Dan Hu, Samuele Notarbartolo, Tom Croonenborghs, Bonny Patel, Ron Cialic, Tun-Hsiang Yang, Dominik Aschenbrenner, Karin M. Andersson, Marco Gattorno, Minh Pham, Pia Kivisakk, Isabelle V. Pierre, Youjin Lee, Karun Kiani, Maria Bokarewa, Emily Tjon, Nathalie Pochet, Federica Sallusto, Vijay K. Kuchroo, Howard L. Weiner

**Affiliations:** 1 Ann Romney Center for Neurologic Diseases and Evergrande Center for Immunologic Diseases, Brigham and Women’s Hospital, Harvard Medical School, Boston, MA 02115 USA; 2 Institute for Research in Biomedicine, Università della Svizzera italiana, via Vincenzo Vela 6, CH-6500 Bellinzona, Switzerland; 3 Program in Translational NeuroPsychiatric Genomics, Brigham and Women’s Hospital, Harvard Medical School, Boston, MA 02115 USA; 4 KU Leuven Technology Campus Geel, AdvISe, Kleinhoefstraat 4, 2440 Geel, Belgium; 5 Broad Institute of Massachusetts Institute of Technology and Harvard, Cambridge, MA 02142 USA; 6 Department of Genetics, Harvard Medical School, Boston, MA 02115 USA; 7 Department of Rheumatology and Inflammation Research, Sahlgrenska University Hospital, Gothenburg University, Box 480, 405 30 Gothenburg, Sweden; 8 Second Division of Pediatrics, G. Gaslini Scientific Institute, Largo Gerolamo Gaslini, 5, 16100 Genova(GE), Italy; 9 Institute of Microbiology, ETH Zurich, Vladimir-Prelog-Weg 1-5/10, 8093 Zürich, Switzerland; 10 Present Address: Translational Gastroenterology Unit, NDM Experimental Medicine, University of Oxford, Headington, OX3 9DU UK

## Abstract

We have previously reported the molecular signature of murine pathogenic T_H_17 cells that induce experimental autoimmune encephalomyelitis (EAE) in animals. Here we show that human peripheral blood IFN-γ^+^IL-17^+^ (T_H_1/17) and IFN-γ^−^IL-17^+^ (T_H_17) CD4^+^ T cells display distinct transcriptional profiles in high-throughput transcription analyses. Compared to T_H_17 cells, T_H_1/17 cells have gene signatures with marked similarity to mouse pathogenic T_H_17 cells. Assessing 15 representative signature genes in patients with multiple sclerosis, we find that T_H_1/17 cells have elevated expression of *CXCR3* and reduced expression of *IFNG*, *CCL3*, *CLL4*, *GZMB*, and *IL10* compared to healthy controls. Moreover, higher expression of *IL10* in T_H_17 cells is found in clinically stable vs. active patients. Our results define the molecular signature of human pro-inflammatory T_H_17 cells, which can be used to both identify pathogenic T_H_17 cells and to measure the effect of treatment on T_H_17 cells in human autoimmune diseases.

## Abstract (web summary)

CD4^+^ T cells secreting interleukin-17 (T_H_17) have diverse functions in modulating autoimmune diseases. Here the authors show via transcriptome analyses that a subset of human T_H_ 17 co-expressing interferon-γ (T_H_1/17) has a molecular signature similar to “pathogenic” mouse T_H_ 17 but distinct from “non-pathogenic” mouse T_H_ 17.

## Introduction

T_H_17 cells are a subset of interleukin-17 (IL-17)-secreting T-helper (T_H_) cells implicated in the pathogenesis of multiple sclerosis (MS), rheumatoid arthritis, juvenile idiopathic arthritis (JIA), and psoriasis^1,2^, whose differentiation is regulated by the transcription factor RAR-related orphan nuclear receptor gamma (RORγt)^3^. Initially, T_H_17 cells were considered a uniformly pro-inflammatory population driven by IL-23 and expressed a unique pattern of pro-inflammatory cytokines different from T_H_1 and T_H_2 cells^4–6^. Subsequent studies showed the function of T_H_17 cells in autoimmune diseases and defense against bacterial and fungal pathogens^7–10^. T_H_17 cells can be induced to produce T_H_1 and T_H_2 cytokines^11^ and not all T_H_17 cells are pathogenic. Murine T_H_17 cells are pathogenic or non-pathogenic based on their ability to induce experimental autoimmune encephalomyelitis (EAE)^12^; pathogenic T_H_17 cells express higher levels of IFN-γ while non-pathogenic T_H_17 cells produce IL-10 with IL-17^13^.

As in mice, human T_H_17 cells can also co-produce IFN-γ or IL-10. IL-10-producing T_H_17 cells are induced in response to *Staphylococcus aureus*, whereas T_H_17 cells induced by *Candida albicans* produce IL-17 and IFN-γ. Both types of T_H_17 cells are enriched in a subset of human memory CD45RA^–^CD4^+^ T_H_ cells expressing the chemokine receptors CCR6 and CCR4, while IFN-γ-secreting T_H_17 (T_H_1/17) cells may additionally express CXCR3^9,14^. A deficiency in IL-17 or the T_H_17 pathway compromises host defenses against *S. aureus* and *C. albicans*, and reduces the frequency of circulating CCR6^+^ memory CD4^+^ T_H_ cells^15,16^. Thus both IFN-γ and IL-10-producing T_H_17 cells may be protective during infection.

IFN-γ and IL-10-producing T_H_17 cells are considered pro-inflammatory and anti-inflammatory, respectively, and have opposite functions in autoimmunity^17,18^. Studies of T-cell libraries from patients with MS showed that CCR6^+^ myelin-reactive T cells exhibit enhanced production of IFN-γ, IL-17, and GM-CSF and reduced production of IL-10, when compared with those from healthy individuals^19^. In JIA, IFN-γ-secreting T_H_1/17 cells are highly enriched in the synovial fluid (SF) of inflamed joints^20^. The inflammatory environment in diseased joints can induce IFN-γ-negative T_H_17 cells to co-produce IFN-γ, implicating plasticity of T_H_17 cells^21^. The proportions of T_H_1/17-enriched CD4^+^CD161^+^ T cells in the SF of affected joints correlate with the erythrocyte sedimentation rate and serum levels of C-reactive protein, suggesting these cells function in disease pathogenicity^20^. These and other reports of elevated numbers of T_H_1/17 cells in inflamed tissues in human autoimmune diseases^21–23^ associate T_H_1/17 cells with human autoimmune diseases.

The complexity of T_H_17 function is further manifested in therapeutic studies. Although anti-IL-17 therapy benefits psoriasis, blocking the IL-17 pathway in Crohn’s disease is either ineffective or exacerbates diseases^24–26^. Similarly, in the CD45RB^hi^ adoptive transfer mouse model of experimental colitis, a deficiency of IL-17 production or IL-17R expression in transferred CD45RB^hi^ CD4 T cells results in accelerated disease^27^. The protective function of IL-17 in these studies may be due to the fact that T_H_17 cells that line the gut mucosa prevent invasion of the gut microbiome and promote intestinal homeostasis^28^. In tumors, T_H_17 cells are reported to have both beneficial^29–31^ and detrimental effects^32^ both in animal models and human disease. Hence, the function of T_H_17 cells in diverse immune responses is complex.

We previously reported that murine T_H_17 cells can be differentiated into pathogenic vs. non-pathogenic subsets, as well as characterized the molecular signature of murine pathogenic T_H_17 cells through global gene expression analysis^12^. In the present study, we compare the gene expression profiles between human IFN-γ^+^ and IFN-γ^–^ T_H_17 subsets, and between IL-10^+^ and IL-10^–^ T_H_17 clones. Comparative transcriptomic analyses show that human T_H_1/17 cells and IL-10^–^ T_H_17 clones display gene signatures with marked similarities to mouse pathogenic T_H_17 cells. We then assess T_H_1/17 cells in patients with MS and find reduced expression of anti-inflammatory *IL10* and elevated expression of *CXCR3*. When we compare clinically active vs. stable patients, we find that stable patients have higher *IL10* expression in T_H_17 cells, whereas active patients have higher expression of *STAT3* in IFN-γ^–^/IL-17^–^ CD4^+^ T cells. Our results define the molecular signature of human pro-inflammatory T_H_17 cells, which can be used to both identify pathogenic T_H_17 cells and to measure the effect of treatment on T_H_17 cells.

## Results

### Transcriptionally distinct T_H_17 subsets in peripheral blood

**Fig. 1:**
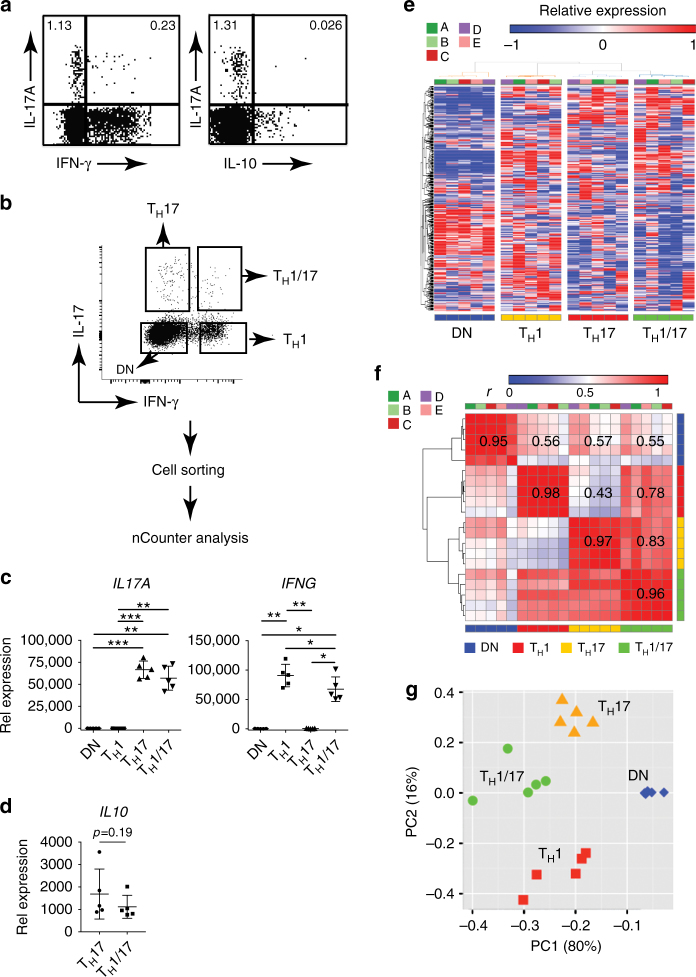
Transcriptionally distinct human T_H_17 subsets in peripheral blood. **a** IFN-γ and IL-10 expression in human T_H_17 cells. Isolated PBMCs were stimulated with PMA and ionomycin for 4 h. Production of indicated cytokines in CD4^+^ T cells were assessed by flow cytometry with intracellular cytokine staining assay. Dotplots shown were gated on CD4^+^ lymphocytes. Data are representative of two independent experiments with similar results. **b** Isolation of live T_H_1/17, T_H_17, T_H_1, and DN cells from human PBMC for nCounter analysis. CD4^+^ T cells isolated from the peripheral blood of healthy donors were stimulated with PMA and ionomycin for 3 h. CD3^+^CD4^+^-T_H_1/17 (IFN-γ^+^IL-17^+^), T_H_17 (IFN-γ^−^IL-17^+^), T_H_1 (IFN-γ^+^IL-17^−^), and DN (IFN-γ^−^IL-17^−^) cells were sorted after being stained with fluorescence-conjugated anti-CD3 and CD4 in combination with cytokine secretion detection kits (Miltenyi) (*n* = 5). **b** Isolated CD4^+^ T subsets were stimulated and stained as in **a**. **c**–**f** CD4^+^ T-cell subsets treated as in **a** were measured using the nCounter (nanoString Technologies) CodeSet HuT_H_17 and subsequently analyzed (hereafter abbreviated as nCounter analysis). **c** Differential expression analysis of mRNA levels of *IL17A* and *IFNG*. *p < 0.05, **p < 0.005, ***p < 0.0005, One-way ANOVA with Tukey’s multiple comparison test (mean ± s.d.). **d** Differential expression analysis of mRNA levels of *IL10*. Two tailed, paired Student’s *t* test *p*-value was shown (mean ± s.d.). The 326 out of the 418 measured genes in the HuT_H_17 CodeSet that showed unsupervised variation across the sample population were used for **e** hierarchical clustering of the individual samples (individual donors: A, B, C, D, and E), **f** hierarchical clustering of the Pearson’s linear correlations between the samples (individuals *n* = 5; individual donors: A, B, C, D, and E), and **g** principal component analysis of the samples (individuals *n* = 5)

We first performed intracellular cytokine staining of blood CD4^+^ T cells and identified distinct populations of IFN-γ co-producing T_H_17 cells, but no IL-10 co-producing T_H_17 cells (Fig. 1a; Supplementary Fig. 1). It is known that IFN-γ^+^ T_H_17 cells are increased in inflamed tissues in human autoimmune diseases^21–23^, and are also present in the blood of healthy individuals, whereas IL-10^+^ T_H_17 cells are barely detected^14^. We divided peripheral T_H_17 cells into IFN-γ^+^ (T_H_1/17) and IFN-γ^–^ (T_H_17) subsets. We utilized a capture assay that separates live CD4^+^ T subsets based on differential secretion of IL-17 and/or IFN-γ to sort ex vivo T_H_1/17 cells and T_H_17 cells without in vitro polarization and with only short-term (3 h) Phorbol 12-myristate 13-acetate (PMA) plus ionomycin stimulation (Fig. 1b; Supplementary Fig. 2). Based on our global transcriptional analysis of murine T_H_17 cells and studies on autoimmunity from ours and other groups, we designed a nanoString nCounter CodeSet HuT_H_17 that detects 418 genes associated with human T_H_ cell differentiation and activation. The HuT_H_17 CodeSet encompasses genes encoding transcription factors, cytokines, cell surface markers, kinases, lytic proteins, and housekeeping proteins (Supplementary Data 1). We used this CodeSet to generate high-throughput transcription profiles of isolated ex vivo T_H_1/17, T_H_17, T_H_1, and double negative (DN) CD4^+^ T cells from five healthy donors to generate high-throughput transcription profiles. We found high expression of *IL17A* in T_H_17 and T_H_1/17 cells and high expression of *IFNG* in T_H_1 and T_H_1/17 cells, whereas only minimal expression of *IL17A* was observed in T_H_1 and DN cells and minimal expression of *IFNG* was observed in T_H_17 and DN cells (Fig. 1c), thus demonstrating that we isolated pure populations of T_H_1/17, T_H_17, and T_H_1 cells. *IL10* gene expression was detected in both T_H_17 and T_H_1/17 cells (Fig. 1d).

For the 20 CD4^+^ T-cell subset samples from the five healthy individuals we analyzed, 362 of the 418 genes demonstrated unbiased variation across the population, defined as an unsupervised expression difference (difference between maximum and minimum relative gene expression values, not taking into account information about the subset classes) ≥5 across all samples, and these genes were selected for further study. Hierarchical clustering of gene expression profiles of the 20 individual samples from the CD4^+^ subpopulations in the context of these 362 unsupervised varying genes properly segregated the T_H_1/17, T_H_17, T_H_1, and DN cells into four different clusters, revealing their distinct transcriptional features (Fig. 1e). The in-group Pearson correlation values for these gene expression profiles were high for all four CD4^+^ T-cell subsets and ranged from 0.95 ± 0.06 for DN cells to 0.98 ± 0.02 for T_H_1 cells. Pearson correlation values for the gene expression profiles of T_H_1/17 vs. T_H_17 and T_H_1/17 vs. T_H_1 were 0.83 ± 0.05 and 0.78 ± 0.06, respectively, whereas the correlation coefficient for T_H_17 vs. T_H_1 was only 0.43 ± 0.06 (Fig. 1f), which were consistent with the degree of similarities observed among the cell subsets in the hierarchical clustering analysis (Fig. 1e). Principal component analysis (PCA) showed that DN cells were clearly distinct from the other three subsets, whereas T_H_1/17 cells lay in between T_H_17 and T_H_1 cells (Fig. 1g). These results demonstrate that human T_H_17 cells can be transcriptionally categorized into IFN-γ^+^ T_H_17 (T_H_1/17) and IFN-γ^–^ T_H_17 (T_H_17) subsets and that T_H_1/17 show a close relationship to both T_H_17 and T_H_1 cells.

### Human ex vivo T_H_17 subsets vs. murine T_H_17 cells

**Fig. 2:**
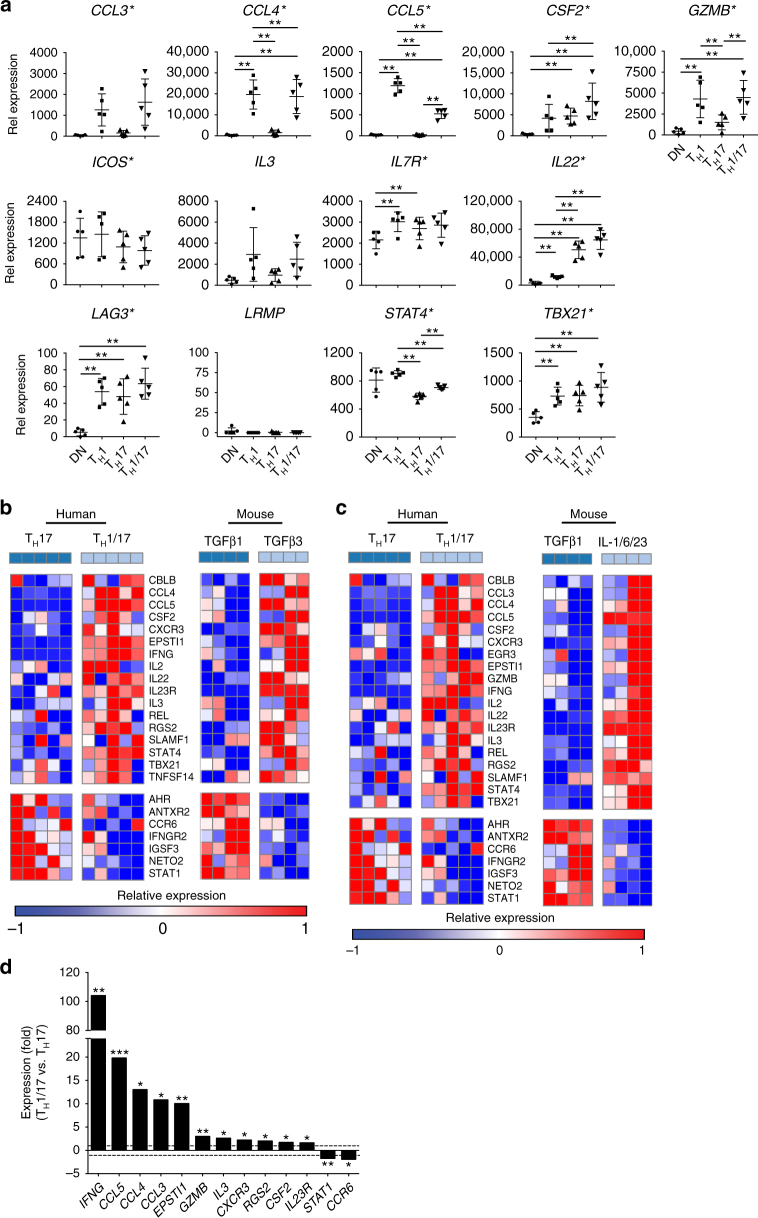
Gene expression comparison between human T_H_1/17 vs. T_H_17 cells and mouse pathogenic vs. non-pathogenic T_H_17 cells. **a** The expression of previously reported murine and human T_H_17 signature genes in purified ex vivo T_H_1 cells. The mRNA gene expression levels in T_H_1/17, T_H_17, T_H_1, and DN cells were measured as described in Fig. 1. *p < 0.05, repeated measures one-way ANOVA; **p < 0.05, pairwised group comparison with Tukey’s multiple comparison test (mean ± s.d., *n* = 5). **b**, **c** Gene set enrichment analysis comparing human T_H_1/17 vs. T_H_17 cells with mouse pathogenic vs. non-pathogenic T_H_17 cells. **b** Heatmap of upregulated (upper panels) and downregulated (lower panels) “leading edge” genes of comparison Scenario 1: human T_H_1/17 vs. T_H_17 cells vs. mouse TGF-β3 plus IL-6-induced T_H_17 cells vs. TGF-β1 plus IL-6-induced T_H_17 cells (Kolmogorov–Smirnov test *p* < 0.0001; FDR *q* < 0.0001 for upregulated genes; Kolmogorov–Smirnov test *p* = 0.0007; FDR *q* = 0.001 for downregulated genes). **c** Heatmap of upregulated (upper panels) and downregulated (lower panels) “leading edge” genes of comparison Scenario 2: human T_H_1/17 vs. T_H_17 cells vs. mouse IL-1, IL-23 plus IL-6-induced T_H_17 cells vs. TGF-β1 plus IL-6-induced T_H_17 cells (Kolmogorov–Smirnov test *p* < 0.0001; FDR *q* < 0.0001 for upregulated genes; Kolmogorov–Smirnov test *p* = 0.004; FDR *q* = 0.004 for downregulated genes). Each column represents one donor in human *(n* = 5) or one sample in murine (*n* = 4). **d** The robust predicted pathogenic signature (PreP-Signature) of human T_H_1/17 cells. Signature genes are those identified as differentially expressed between human T_H_1/17 and T_H_17 cells that are identified as enriched “leading edge” genes when assessing these human genes in the mouse profiles in **b**, **c** and that are additionally curated for robustness based on supervised absolute fold change >1.5. Two tailed, paired Student’s *t* test *p*-value < 0.05. *p < 0.05, **p < 0.05, ***p < 0.0005, *n* = 5

Murine T_H_17 cells generated with TGF-β3/IL-6 or IL-1/IL-6/IL-23 induce more severe EAE than T_H_17 cells generated with TGF-β1/IL-6^12^. In comparing TGF-β3 vs. TGF-β1-induced murine T_H_17 cells, we defined a pathogenic transcriptional signature composed of 16 upregulated genes in TGF-β3-induced pathogenic T_H_17 cells including *CCL3, CCL4, CLL5, CSF2, IL22, IL3, GZMB, STAT4*, and *TBX21* ^12^. In humans, 10 of the 16 signature genes were upregulated in CCR6^+^ myelin-reactive memory CD4^+^ T cells in patients with MS although these cells also displayed elevated levels of *IL10* ^19^, while 12 genes were upregulated in T_H_1/17-enriched CCR7^lo^CCR6^+^CCR4^lo^CXCR3^hi^ memory CD4^+^ T cells in healthy donors compared to T_H_17-enriched CCR7^lo^CCR6^+^CCR4^hi^CXCR3^lo^ cells^33^ (Supplementary Data 2). There are no known unique surface markers for T_H_17 cells and all of memory CD4^+^ T-cell populations contain not only IFN-γ^+^ and IFN-γ^–^ T_H_17 cells but also IL-17^–^IFN-γ^+^ T_H_1 cells, especially for CCR7^lo^CCR6^+^CCR4^lo^CXCR3^hi^ memory CD4^+^ T cells, for which the frequency of IL-17^–^IFN-γ^+^ cells may be up to four times higher than IL-17^+^ cells^33^. Among the 14 signature genes shared by murine T_H_17 cells and human T_H_17-enriched CD4^+^ T cells, 13 are included in the HuT_H_17 CodeSet (Supplementary Data 2) and we assessed their expression in T_H_1/17, T_H_17, T_H_1, and DN CD4^+^ T cells (Fig. 2a). We found that except for *LRMP*, the other 12 genes were detected by the CodeSet. Compared to T_H_1/17 cells, 10 of the 12 genes had comparable or higher messenger RNA (mRNA) levels in T_H_1 cells, which included *CCL3, CCL4, CCL5, GZMB, ICOS, IL3, IL7R, LAG3, STAT4*, and *TBX21*, emphasizing the importance of minimizing T_H_1 contamination when analyzing the gene signature of pro-inflammatory or pathogenic T_H_17 cells. The high purity of the T_H_1/17 and T_H_17 cells we isolated based on IL-17 and IFN-γ secretion allowed us to determine the pathogenicity-associated molecular signature of human T_H_17 subsets.

We identified 60 genes differentially expressed in T_H_1/17 cells vs. T_H_17 cells with 39 upregulated and 21 downregulated (Supplementary Data 3). To test the hypothesis that the relationship of T_H_1/17 to T_H_17 cells in humans is similar to that of mouse pathogenic vs. non-pathogenic T_H_17 cells, we performed gene set enrichment analysis (GSEA)^34^. The upregulated and downregulated genes in T_H_1/17 cells relative to T_H_17 cells were divided into two gene sets. Since both TGF-β3/IL-6 and IL-1/IL-6/IL-23 induced mouse pathogenic T_H_17 cells whereas TGF-β1/IL-6 induced non-pathogenic T_H_17 cells^12^, our first comparison (Scenario I) explored microarray data from TGF-β3/IL-6-induced T_H_17 cells (pathogenic) vs. TGF-β1/IL-6-induced T_H_17 cells (non-pathogenic) and the second comparison (Scenario II) explored microarray data from IL-1/IL-6/IL-23-induced T_H_17 cells (pathogenic) vs. TGF-β1/IL-6-induced T_H_17 cells (non-pathogenic). GSEA results demonstrated that genes upregulated in human T_H_1/17 vs. T_H_17 cells were enriched in mouse pathogenic T_H_17 cells vs. non-pathogenic T_H_17 cells in both scenarios with 17 “leading edge” genes in Scenario 1 and 19 genes in Scenario 2 (Kolmogorov–Smirnov test *p* < 0.0001; false discovery rate (FDR) *q* < 0.0001 for both scenarios) (upper panels of Fig. 2b, c). The “leading edge” subset of genes is defined as genes that drive the enrichment scores, thus the genes that appear in the top of the ranked list of genes at, or before, the point where the running sum reaches the maximum deviation from zero. The “leading edge” subset can be interpreted as the core subset of a gene set that accounts for the enrichment signal^34^. Genes upregulated in human T_H_17 vs. T_H_1/17 cells were also enriched in mouse non-pathogenic T_H_17 cells vs. pathogenic T_H_17 cells in both comparison scenarios, with seven identical “leading edge” genes (Kolmogorov–Smirnov test *p* = 0.0007; FDR *q* = 0.001 for Scenario I; Kolmogorov–Smirnov test *p* = 0.004; FDR *q* = 0.004 for Scenario II) (lower panels of Fig. 2b, c). The gene signatures obtained from both scenarios were almost identical even though the TGF-β3/IL-6-induced T_H_17 cells and IL-1/IL-6/IL-23-induced T_H_17 cells were differentiated via treating naive CD4^+^ T cells with different cytokines, suggesting that the integrated gene expression analysis identified common features shared by the two types of mouse pathogenic T_H_17 cells. Thus, we found marked similarities in differential gene expression signatures between human IFN-γ^+^ vs. IFN-γ^–^ T_H_17 cells and mouse pathogenic vs. non-pathogenic T_H_17 cells indicating that human T_H_1/17 and T_H_17 cells are counterparts of murine pathogenic and non-pathogenic T_H_17 cells.

Among the 27 signature genes identified in both comparison scenarios, 13 had an absolute fold change >1.5 for T_H_1/17 vs. T_H_17 cells, which were selected as the robust predicted pathogenic signature (PreP-Signature) of T_H_1/17 cells for later analyses (Fig. 2d). The upregulated robust PreP-Signature genes with pro-inflammatory/pathogenic potential can be grouped into chemokines and cytokines (*CCL3*, *CCL4*, *CCL5*, *CSF2*, *IFNG*, and *IL3*), chemokine and cytokine receptors (*CXCR3* and *IL23R*), cytokine responding genes (*EPSTI1*), effector proteins (*GZMB*), and signaling molecules (*RGS2*). The downregulated genes are the chemokine receptor (*CCR6*) and the transcription factor (*STAT1*).

We also investigated genes not shared between human and mouse pathogenic/non-pathogenic Th17 cells to identify other potentially relevant genes to test in human conditions. We identified 33 non-shared genes including 19 that were upregulated and 14 that were downregulated (Supplementary Data 4).

### Human ex vivo T_H_17 subsets vs. T_H_17 clones

**Fig. 3:**
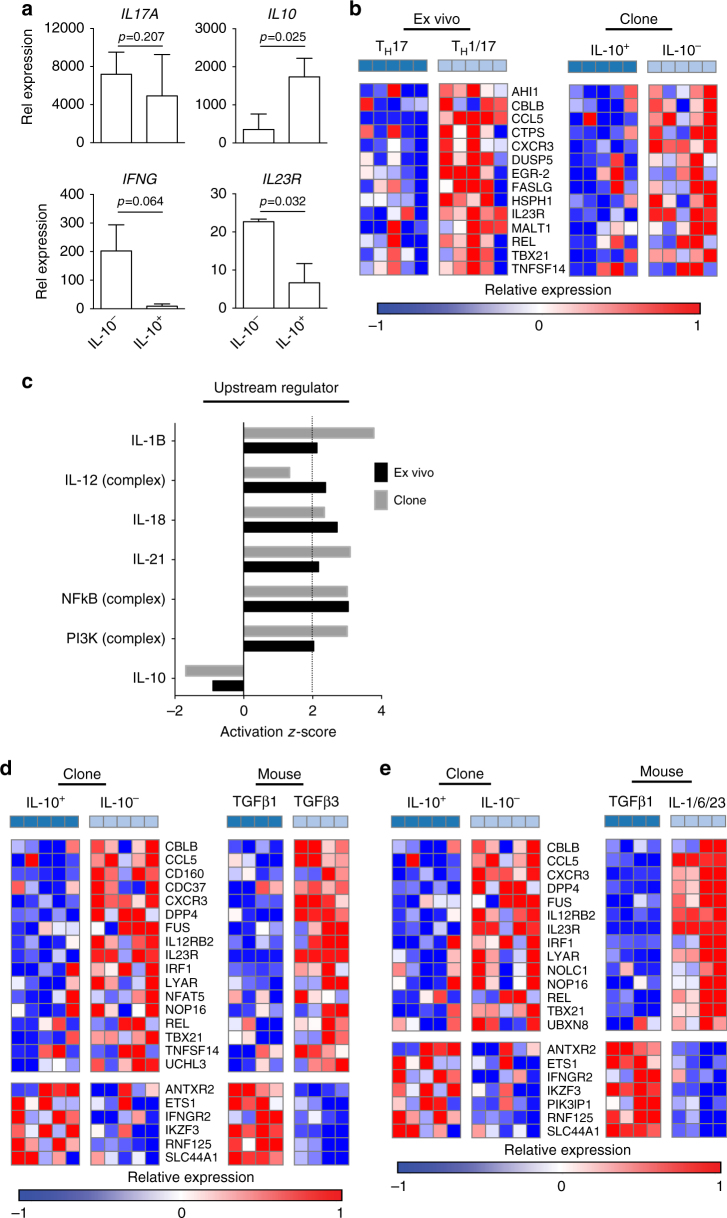
Gene expression comparison between human T_H_1/17 vs. T_H_17 cells and IL-10^–^ vs. IL-10^+^ T_H_17 clones. **a** Quantitative RT-PCR analysis of gene expression in human IL-10^–^ and IL-10^+^ T_H_17 clones isolated from healthy donors (two tailed, paired Student’s *t* test, mean ± s.d., *n* = 3). For *IFNG*, *IL17A*, and *IL23R*, resting T_H_17 clones were stimulated with anti-CD3 and anti-CD28 for 4 h before RNA extraction. For *IL10*, resting T_H_17 clones were stimulated with anti-CD3 and anti-CD28 for 5 days, then cells were re-stimulated with anti-CD3 and anti-CD28 for 4 h before RNA extraction. **b** Hypergeometric enrichment test between human T_H_1/17 vs. T_H_17 cells and IL-10^–^ vs. IL-10^+^ T_H_17 clones. Genes differentially expressed between human T_H_1/17 and T_H_17 cells (Supplementary Data 3) were analyzed for enrichment in those of human IL-10^–^ vs. IL-10^+^ T_H_17 clones (Supplementary Data 5). Heatmap shows the overlapping genes (one-sided Fisher’s exact test *p* < 0.0001, FDR *q* = 0.0001). Each column represents one donor (*n* = 5). **c** Predicted upstream regulators for T_H_1/17 and IL-10^–^ T_H_17 clone differentiation. The differentially expressed genes with corresponding fold changes and *p*-values from the T_H_1/17 vs. T_H_17 comparison (Supplementary Data 3) and IL-10^–^ vs. IL-10^+^ T_H_17 clone comparison (Supplementary Data 5) were analyzed using the IPA upstream regulator analysis. Ex vivo, T_H_1/17 vs. T_H_17 comparison; Clone, IL-10^–^ vs. IL-10^+^ T_H_17 clone comparison. **d**, **e** Gene set enrichment analysis comparing human IL-10^–^ vs. IL-10^+^ T_H_17 clones with mouse pathogenic vs. non-pathogenic T_H_17 cells. **d** Heatmap of upregulated (upper panels) and downregulated (lower panels) “leading edge” genes of comparison Scenario 1: human IL-10^–^ vs. IL-10^+^ T_H_17 clones vs. mouse TGF-β3 plus IL-6-induced T_H_17 cells vs. TGF-β1 plus IL-6-induced T_H_17 cells (Kolmogorov–Smirnov test *p* = 0.004; FDR *q* = 0.005 for upregulated genes; Kolmogorov–Smirnov test *p* = 0.004; FDR *q* = 0.003 for downregulated genes). **e** Heatmap of upregulated (upper panels) and downregulated (lower panels) ‘‘leading edge” genes of comparison Scenario 2: human IL-10^–^ vs. IL-10^+^ T_H_17 clones vs. mouse IL-1, IL-23 plus IL-6-induced T_H_17 cells vs. TGF-β1 plus IL-6-induced T_H_17 cells (Kolmogorov–Smirnov test *p* = 0.005; FDR *q* = 0.010 for upregulated genes; Kolmogorov–Smirnov test *p* = 0.016; FDR *q* = 0.009 for downregulated genes). Each column represents one donor in human (*n* = 5) or one sample in murine (*n* = 4)

IL-10 has a pivotal role in regulation of both innate and adaptive immunity^35^ and murine TGF-β1/IL-6-induced non-pathogenic T_H_17 cells produce IL-10^13^. Human IL-10^+^ T_H_17 cells are a potential counterpart of mouse non-pathogenic T_H_17 cells. However, IL-10-secreting T_H_17 cells cannot be directly isolated from human blood for nCounter gene expression analysis because few or no T_H_17 cells produce IL-10 after PMA/ionomycin stimulation (Fig. 1a). This is likely due to the delayed production of IL-10 after stimulation. Nonetheless, for established human T_H_17 clones, IL-10^+^ T_H_17 clones can be identified by IL-10 intracellular staining 5 days after T-cell receptor activation^9^. Thus, we established CD4^+^ T cells clones from CCR6^+^CCR4^+^CXCR3^–^ memory CD4^+^ T cells (Supplementary Fig. 3) that were enriched for T_H_17 cells^14,18^ and screened for clones producing IL-17 with or without co-secretion of IL-10. qRT-PCR showed that IL-10^–^ T_H_17 clones expressed high levels of *IFNG*, low levels of *IL10*, and high levels of *IL23R*, whereas IL-10^+^ T_H_17 clones expressed minimal levels of *IFNG* and high levels of *IL10* (Fig. 3a). To assess whether the gene expression profile of cloned T_H_17 cells maintained their pro or anti-inflammatory features after long-term culture, we used the HuT_H_17 CodeSet to analyze the gene expression profile of IL-10^–^ and IL-10^+^ T_H_17 clones. We identified 63 genes that were differentially expressed between IL-10^–^ vs. IL-10^+^ T_H_17 clones with 49 upregulated and 14 downregulated genes (Supplementary Data 5). To compare genes differentially expressed between human T_H_1/17 vs. T_H_17 cells (Supplementary Data 3) to those between IL-10^–^ vs. IL-10^+^ T_H_17 clones (Supplementary Data 5), we assessed enrichment of overlaps between gene lists using the hypergeometric enrichment test. We found that genes upregulated in T_H_1/17 vs. T_H_17 cells display significant overlap with genes upregulated in IL-10^–^ vs. IL-10^+^ T_H_17 clones (one-sided Fisher’s exact test *p* < 0.0001, FDR *q* = 0.0001) (Fig. 3b). The upregulation of *CBLB, CLL5, CXCR3, IL23R, REL, TBX21*, and *TNFSF14* in IL-10^–^ T_H_17 clones is shared by human T_H_1/17 cells and mouse pathogenic T_H_17 cells (Fig. 2b, c). To identify regulatory molecules predicted to influence the differentiation/development of T_H_1/17 cells and IL-10^–^ T_H_17 clones, we interrogated genes differentially expressed between T_H_1/17 vs. T_H_17 cells (Supplementary Data 3) and between IL-10^–^ vs. IL-10^+^ T_H_17 clones (Supplementary Data 5) for upstream regulator prediction analysis in ingenuity pathway analysis (IPA). T_H_1/17 cells and IL-10^–^ T_H_17 clones displayed a similar pattern of the activation of signaling pathways involved in T_H_17 differentiation/development (Fig. 3c), especially for activation of IL-1β signaling, which is critical to promote co-producing IFN-γ and to inhibit IL-10 production in T_H_17 cells^9^. These results demonstrate the similarities between T_H_1/17 vs. T_H_17 cells and IL-10^–^ vs. IL-10^+^ T_H_17 clones, and also indicate that IL-10^–^ and IL-10^+^ T_H_17 clones maintained their pro- or anti-inflammatory characteristics after long-term culture.

### Human T_H_17 clones vs. murine T_H_17 cells

We performed GSEA to investigate the similarity between human T_H_17 clones and mouse T_H_17 cells. The upregulated and downregulated genes in IL-10^–^ T_H_17 clones relative to IL-10^+^ T_H_17 clones T_H_17 cells were divided into two gene sets (Supplementary Data 5) to explore the enrichment of the gene sets in Scenarios I and II (defined above). GSEA showed that genes upregulated in IL-10^–^ vs. IL-10^+^ T_H_17 clones were enriched in mouse pathogenic vs. non-pathogenic T_H_17 cells in both scenarios, with 17 “leading edge” genes in Scenario I (Kolmogorov–Smirnov test *p* = 0.004; FDR *q* = 0.005) and 14 “leading edge” genes in Scenario II (Kolmogorov–Smirnov test *p* = 0.005; FDR *q* = 0.010). Among the “leading edge” genes, 11 overlapped between both scenarios (upper panels of Fig. 3d, e). Genes upregulated in human IL-10^+^ vs. IL-10^–^ T_H_17 clones were also enriched in mouse non-pathogenic vs. pathogenic T_H_17 cells in both scenarios, with six “leading edge” genes in Scenario I (Kolmogorov–Smirnov test *p* = 0.004; FDR *q* = 0.003) and seven “leading edge” genes in Scenario II (Kolmogorov–Smirnov test *p* = 0.016; FDR *q* = 0.009). Among the “leading edge” genes, six genes were represented in both scenarios (lower panels of Fig. 3d, e). These results demonstrate extensive similarities between human IL-10^–^ vs. IL-10^+^ T_H_17 clones and mouse pathogenic vs. non-pathogenic T_H_17 cells.

### T_H_17 PreP-signatures predict STAT3 as an upstream regulator

**Fig. 4:**
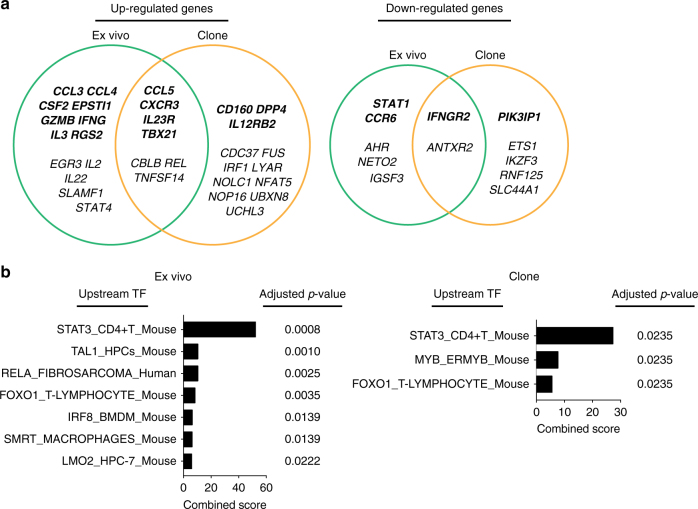
Predicting STAT3 as upstream transcription factor by the PreP-Signatures of T_H_17 cells. **a** Venn diagram representations of signature genes upregulated (left) and downregulated (right) in human T_H_1/17 cells and IL-10^–^ T_H_17 clones. Complete PreP-Signatures from GSEA comparison Scenarios I and II were merged for ex vivo cells (Fig. 2b, c) and T_H_17 clones (Fig. 3d, e), respectively. Genes with supervised absolute fold change >1.5 in either ex vivo cells or T_H_17 clones were shown in italic bold letters. Ex vivo, differentially expressed “leading edge” genes from T_H_1/17 vs. T_H_17 GSEA comparisons presented in green circles; Clone, differentially expressed “leading edge” genes from IL-10^–^ vs. IL-10^+^ T_H_17 clone GSEA comparisons presented in yellow circles. **b** Predicted upstream transcription factors for T_H_1/17 and IL-10^–^ T_H_17 clone differentiation. The molecular signatures of T_H_1/17 cells and IL-10^–^ T_H_17 clones in **a** were analyzed using the Enrichr ChEA2016 analysis and the predicted transcription factors with Benjamini–Hochberg adjusted *p*-value <0.05 were shown. TF transcription factor

Integrated analysis of gene expression profiles of human ex vivo T_H_17 cells, human T_H_17 clones, and cytokine-induced mouse T_H_17 cells resulted in four sets of gene signatures associated with the pathogenicity of T_H_17 cells (Figs. 2, 3). We merged the “leading edge” genes from the above GSEA comparisons and consolidated these four gene signatures into one complete PreP-Signature for human ex vivo T_H_17 cells with 27 signature genes and one complete PreP-Signature for human T_H_17 clones with 26 signature genes. Among the signature genes upregulated in human T_H_1/17 cells and IL-10^–^ T_H_17 clones, seven genes were shared by both types of T_H_17 cells, while for downregulated genes, two genes overlapped between T_H_1/17 cells and IL-10^–^ T_H_17 clones (Fig. 4a). Since our molecular signatures derive from integrated analysis of pro-inflammatory human T_H_17 cells with mouse pathogenic T_H_17 cells in autoimmunity, they reduce the number of potential targeting genes and also help define human T_H_17 cells with potential pathogenicity in autoimmunity.

We interrogated the complete PreP-Signatures of ex vivo T_H_1/17 cells and IL-10^–^ T_H_17 clones for upstream transcription factor prediction analysis using the Enrichr ChEA2016 analysis^36,37^. STAT3 in CD4^+^ T cells was the top predicted transcription factor common for both resulting lists (Fig. 4b; Supplementary Datas 6, 7) and thus may regulate the pathogenicity of human T_H_17 cells in autoimmune diseases.

### Investigation of PreP-Signature genes in multiple sclerosis

**Fig. 5:**
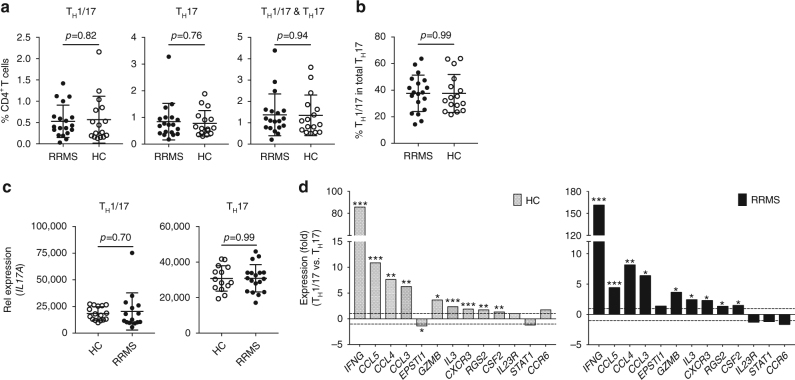
Frequency of T_H_17 subsets and differential expression of PreP-Signature genes in T_H_1/17 vs. T_H_17 cells in MS. Peripheral CD4^+^ T cells isolated from the PBMC of untreated RRMS patients (*n* = 19) and age- and sex-matched healthy controls (HC) (*n* = 16) were stimulated, stained, and sorted for T_H_1/17, T_H_17, T_H_1, and DN cells as described in Fig. 2b. RNA isolated from sorted cell subsets was subjected to low-input qPCR analysis. **a** Frequencies of T_H_1/17, T_H_17, and total T_H_17 cells in total peripheral CD4^+^ T cells (Welch’s *t* test, *p*-values, mean ± s.d.). **b** Frequency of T_H_1/17 in total T_H_17 cells (Welch’s *t* test, mean ± s.d.). **c**, **d** qPCR analysis of gene expression in isolated CD4^+^ T-cell subsets. **c** Comparison of *IL17A* expression between HC and patients in T_H_1/17 or T_H_17 cells (Welch’s *t* test, mean ± s.d.). **d** Differential expression of PreP-Signature genes between T_H_1/17 vs. T_H_17 cells in HC and MS patients. Two tailed, paired Student’s *t* test, *p < 0.05, **p < 0.001, ***p < 0.0001

To investigate PreP-Signature genes in human pro-inflammatory T_H_17 cells in MS, we isolated T_H_1/17 and T_H_17 cells from untreated patients with relapse-remitting MS (RRMS) and age and sex-matched healthy controls (Table 1; Supplementary Fig. 2), assessed frequencies of T_H_1/17 and T_H_17 cells, and measured the expression of the PreP-Signature genes. T_H_1 and DN cells were isolated in parallel as internal controls. Of 19 patients with RRMS, sufficient RNA for quantitative PCR (qPCR) analysis was obtained from 15 T_H_1/17 samples, 18 T_H_17 samples, 19 T_H_1 samples, and 19 DN samples. Of 16 healthy controls, we obtained sufficient RNA for qPCR analysis from 16 T_H_1/17 samples, 14 T_H_17 samples, 16 T_H_1 samples, and 16 DN samples. We found no difference in frequency of T_H_1/17, T_H_17, and total T_H_17 cells among total CD4^+^ T cells in patients vs. controls (Fig. 5a). The percentage of T_H_1/17 in total T_H_17 cells was also similar (Fig. 5b). To assess purity of isolated cell subsets, we measured the expression of *IL17* and *IFNG* by qPCR. We detected high levels of *IL17* in T_H_1/17 and T_H_17 cells but not in DN cells (Supplementary Fig. 4a), and high levels of *IFNG* in T_H_1/17 cells but not in T_H_17 and DN cells (Supplementary Fig. 4b), demonstrating that the isolated populations were of high purity. In both groups, *IL17* expression was higher in T_H_17 vs. T_H_1/17 cells (Supplementary Fig. 4a), whereas *IL17* expression was similar for T_H_1/17 and T_H_17 cells (Fig. 5c). To validate the 13 robust PreP-Signature genes identified via the nCounter analysis, we measured their expression in T_H_1/17 and T_H_17 cells in healthy controls by qPCR (Fig. 5d, left). For the 11 genes upregulated in T_H_1/17 cells, 9 were confirmed; the 2 downregulated genes were not validated. In MS, the nine validated genes displayed the similar upregulated expression pattern (Fig. 5d, right). Thus, the transcriptional regulation of the nine validated robust PreP-Signature genes of T_H_1/17 cells was tightly associated with IFN-γ secretion in both controls and MS. These results indicate that studying frequency or number of IFN-γ-secreting T_H_17 cells may not identify important biological differences.

**Table 1:** Demographics of patients with multiple sclerosis and healthy controls

	RRMS patients	Healthy controls
Participants, *n*	19	16
Gender f/m, *n*	15/4	13/3
Female		
Age, y	46 ± 12	48 ± 11
Male		
Age, y	41 ± 9	42 ± 8
Disease duration, y	10 ± 10	n.a.
EDSS	2.0 ± 1.2	n.a.

*EDSS* expanded disability status scale ranging from 0 to 10, *n.a*. not applicable, *RRMS* untreated relapsing-remitting multiple sclerosis

**Fig. 6:**
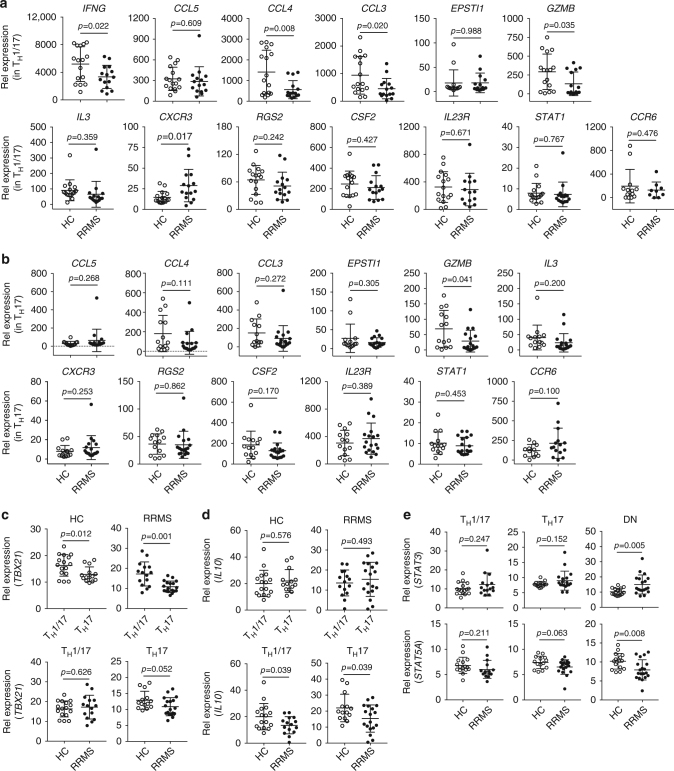
Expression of PreP-signature genes of human pro-inflammatory T_H_17 cells in RRMS. RNA isolated from T_H_1/17, T_H_17, and DN cells (Fig. 5) was subjected to qPCR analysis. Comparison of PreP-Signature gene expression between HC and patients with RRMS in **a** T_H_1/17 cells and **b** T_H_17 cells. (Welch’s *t* test, mean ± s.d.). Comparison of **c** *TBX21* and **d** *IL10* expression between HC and patients with RRMS in T_H_1/17 and T_H_17 cells. **e** Comparison of *STAT3* (upper panel) and *STAT5A* (lower panel) expression between HC and patients with RRMS in T_H_1/17, T_H_17, and DN cells. Welch’s *t* test *p*-values were shown (mean ± s.d.)

We investigated differences between MS and control using the robust PreP signature genes we identified above. We first measured the expression of the 13 robust PreP-Signature genes in T_H_1/17 cells in MS and found elevated expression of *CXCR3* and reduced expression of *IFNG*, *CCL3*, *CLL4*, and *GZMB* (Fig. 6a). In T_H_17 cells, *GZMB* showed reduced expression in MS (Fig. 6b). No difference was observed between MS and controls in T_H_1 cells (Supplementary Fig. 5). Thus, the altered expression of these five PreP-Signature genes are specific for T_H_1/17 and T_H_17 subsets, but not for T_H_1 cells. We then measured the expression of *TBX21* and *IL10*, since we found downregulation of *TBX21* and upregulation of *IL10* in IL-10^+^ T_H_17 clones (Fig. 3a, d). We found *TBX21* expression elevated in T_H_1/17 relative to T_H_17 cells in MS and controls (Fig. 6c, upper panel), with no difference in *IL10* expression (Fig. 6d, upper panel). However, when we compared T_H_1/17 and T_H_17 cells between MS and controls, *IL10* was reduced in T_H_1/17 and T_H_17 cells in patients (Fig. 6d, lower panel), though no difference was detected with *TBX21* expression (Fig. 6c, lower panel). The predicted upstream transcription factor *STAT3* was elevated in the DN cells in MS (Fig. 6e, upper panel), while the expression of its antagonistic transcription factor *STAT5A* ^38–40^ was reduced (Fig. 6e, lower panel).

Thus, the altered expression of five PreP-Signature genes for T_H_1/17 cells in MS (*CXCR3*, *IFNG*, *CCL3*, *CLL4*, and *GZMB*) is T_H_17-specific. The low expression of *IL10* in CXCR3^hi^ T_H_1/17 cells in MS suggests these pro-inflammatory cells may more readily migrate to central nervous system (CNS) since CXCL10 (IP-10), a ligand for CXCR3, is increased in the inflamed CNS in MS^41–44^. It has been suggested that STAT3 facilitates T_H_17 differentiation and STAT5 facilitates Treg differentiation^38–40^, which is consistent with our observation of their expression in DN cells in MS.

**Fig. 7:**
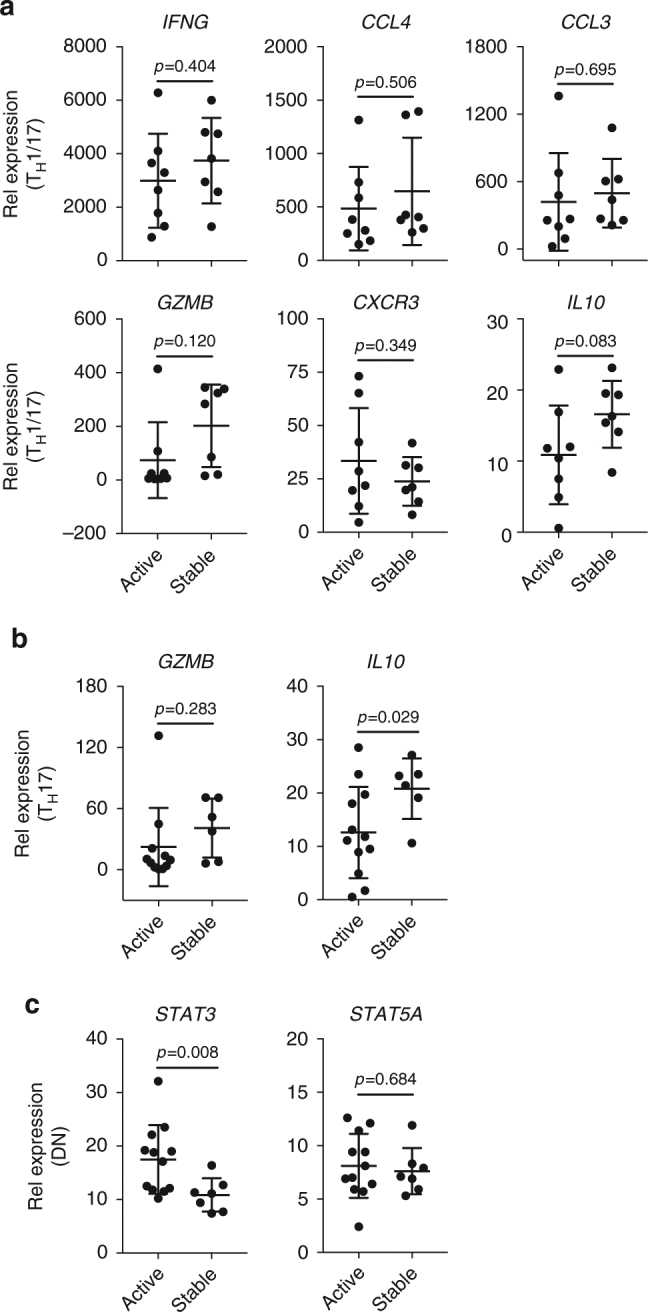
Correlation of *IL10* and *STAT3* expression with disease activity in MS. The mRNA levels of signature genes with altered expression in MS (Fig. 6) were compared between active and stable patients. Signature gene expression in **a** T_H_1/17 (active, *n* = 8; stable, *n* = 7), **b** T_H_17 (active, *n* = 12; stable, *n* = 6), and **c** DN cells (active, *n* = 12; stable, *n* = 7) (Welch’s *t* test, mean ± s.d.)

We then investigated whether the PreP-Signature genes, which had altered expression in MS, were linked to disease activity in MS. Disease activity was defined as a gadolinium-enhancing lesion on magnetic resonance imaging (MRI) or disease relapse within 6 months to the time of blood sampling. All patients were untreated at the time of blood sampling. From 12 active patients, we obtained sufficient RNA for qPCR from 8 T_H_1/17 samples, 12 T_H_17 samples, and 12 DN samples. From seven stable patients, we obtained sufficient RNA for qPCR from 7 T_H_1/17 samples, 6 T_H_17 samples, and 7 DN samples. We found reduced expression of *IL10* in T_H_17 cells (primarily in T_H_1/17 cells) and increased expression of *STAT3* in DN cells in active patients with RRMS (Fig. 7). Given the facilitating role of *STAT3* in T_H_17 differentiation and the anti-inflammatory function of IL-10, these results together with the altered expression of *CXCR3*, *IFNG*, *CCL3*, *CLL4, GZMB*, *IL10*, *STAT3*, and *STAT5A* in MS (Fig. 6) are consistent with an important role for T_H_17 cells in MS.

## Discussion

We found human IL-17-secreting CD4^+^ helper T cells were distinct from T_H_1 cells, and could be transcriptionally segregated into IFN-γ^+^ T_H_17 (T_H_1/17) and IFN-γ^–^ T_H_17 (T_H_17) subsets. On a transcriptional level, T_H_1/17 cells have features of both T_H_17 and T_H_1 cells. In addition to IFN-γ, T_H_1/17 cells expressed elevated levels of the pro-inflammatory molecules CCL3, CCL4, CCL5, granzyme B, IL-3, IL-22, GM-CSF, STAT1, T-bet, and IL-23R, which are pathogenic signature genes of mouse T_H_17 cells^12^. These results not only transcriptionally segregate human T_H_1/17 cells from other T_H_17 cells, but also indicate that these cells have acquired pro-inflammatory properties similar to murine pathogenic T_H_17 cells. This provided the foundation for the cross-species comparative transcriptomic analysis between mouse and human.

We compared the gene expression profiles of human T_H_1/17 cells vs. T_H_17 cells with mouse pathogenic vs. non-pathogenic T_H_17 cells via GSEA and found human T_H_1/17 vs. T_H_17 cells displayed gene expression signatures that were enriched in mouse pathogenic vs. non-pathogenic T_H_17 cells. We obtained similar results in comparing human IL-10^–^ vs. IL-10^+^ T_H_17 clones with and mouse pathogenic vs. non-pathogenic T_H_17 cells. Through this integrated analysis, we identified gene expression features to support the hypothesis that T_H_1/17 cells are the pathogenic T_H_17 population in immune-mediated human disease.

The PreP-Signature genes derived from comparative transcriptional analysis of human and mouse studies identify genes shared between human T_H_1/17 vs. T_H_17 cells or between human IL-10^–^ vs. IL-10^+^ T_H_17 clones and mouse pathogenic vs. non-pathogenic T_H_17 cells. We obtained a robust PreP-Signature for T_H_1/17 cells of 13 genes and assessed them in MS. We find that T_H_1/17 cells in patients with MS have elevated expression of *CXCR3* and reduced expression of *IL10*. It has been shown that CXCL10 (IP-10), a ligand for CXCR3, is increased in the inflamed CNS of MS^41–44^. Given that IL-10 is a potent anti-inflammatory cytokine, this combination may facilitate migration of more pro-inflammatory CXCR3^hi^IL-10^low^ T_H_1/17 cells to the inflamed CNS. However, it is unexpected to observe reduced expression of signature genes *IFNG*, *CCL3*, *CLL4*, and *GZMB* in T_H_1/17 cells in MS vs. healthy controls, especially the reduced expression of *IFNG*. Natalizumab, a humanized monoclonal antibody targeting α4 integrin used in the treatment of MS, functions by preventing immune cells from crossing the blood–brain barrier^45^. A study has shown that both natalizumab treated and untreated patients during relapse have lower frequencies of T_H_17 cells in peripheral blood compared to stable patients. Moreover, T_H_17 cells become almost undetectable in patients with breakthrough disease that occurs following natalizumab withdrawal^46^. Thus, one explanation for reduced expression of *IFNG*, *CCL3*, *CLL4*, and *GZMB* in T_H_1/17 cells in MS could be that migration of cells to the CNS removes CXCR3 high, active T_H_1/17 cells from the blood. Another possibility may be that expression of IFN-γ ensures high expression of CXCR3, which is critical for cells to migrate to inflamed CNS, and reduced IFN-γ may indicate the further enhancement of the pathogenicity of IL-10^lo^ T_H_17 cells in MS. IFN-γ-stimulation is required for CXCR3 induction on T cells upon T-cell receptor stimulation^47^. Although counter-intuitive, the potent pro-inflammatory T_H_1 cytokine IFN-γ is protective during EAE induction^48–50^. It suppresses EAE induction by inhibiting generation of T_H_17 cells^51^, converting CD4^+^CD25^–^ T cells to CD4^+^ Tregs^52^ and limiting myelin lipid peroxidation in CNS^53^. IFN-γ is dispensable for generation of pathogenic T_H_17 cells, however, T-bet the transcription factor for T_H_1 was initially considered essential due to the high resistance of Tbet^−/−^ mice to EAE^54,55^. Later studies show that T-bet is essential for T_H_1 but not T_H_17-mediated EAE^56,57^. Studies in Tbet^−/−^ mice have shown that reduced IFN-γ in T_H_17 cells does not affect their pathogenicity though the conversion of T_H_17 cells to T_H_1/17 cells as well as T_H_1-like IFN-γ ^+^ ex-T_H_17 cells is prevented^56,58,59^.

Interrogating the PreP-Signatures for the identification of upstream regulators and transcription factors^36,37^, we identified STAT3 as the top predicted upstream transcription factor from previous studies in CD4^+^ T cells, which suggests that STAT3 may regulate the pathogenesis of human T_H_17 cells in autoimmune diseases. It has been reported that STAT3 is a critical regulator for the induction of T_H_17 cells in humans. Humans with a genetic defect in STAT3 expression not only have reduced expression of T_H_17 without any impact on other T-cell subsets, but also develop a hyper-IgE syndrome with severe infections of *C. albicans* and *S. aureus* ^60,61^. STAT3 and STAT5 are a pair of mutual restraint transcription factors that regulate T_H_17 and Treg differentiation with STAT3 facilitates T_H_17 differentiation and STAT5 facilitates Treg differentiation^38–40^. We thus investigated the expression of STAT3 and STAT5A in MS. We found upregulation of *STAT3* and downregulation of *STAT5A* in DN cells from patients with MS, which included naive and other memory CD4^+^ T cells but not T_H_1/17, T_H_17, and T_H_1 cells. One explanation for the different behavior of STAT3 and STAT5A between DN vs. T_H_1/17 and T_H_17 cells is that STAT3 may be critical for T_H_17 differentiation but not required for T_H_17-associated immunopathology after differentiation.

Leveraging comparative transcriptomic approaches, we integrated gene expression profiles derived from human and mouse T_H_17 cells to identify pathogenicity-associated signature (PreP-Signature) genes that are shared by human pro-inflammatory T_H_17 cells and mouse pathogenic T_H_17 cells, and to predict the upstream regulators or transcription factors that may be critical for the differentiation of human pro-inflammatory T_H_17 cells. These comparative transcriptomic analyses allowed us to identify altered gene expression associated with T_H_17 subsets and their differentiation in subjects with MS and to identify associations of the expression of *STAT3* in DN cells and *IL10* in T_H_17 cells that are dependent on MS disease activity. Of note, we used a nCounter codeset with a limited set of pre-selected 418 genes, which may miss other disease related, highly discriminant genes in humans. Thus, follow-up studies, such as RNA-sequencing analysis on T_H_17 and T_H_1/17 subsets isolated from MS patients, may help to identify more disease-related genes.

In summary, our study demonstrates that human T_H_1/17 cells and IL-10^−^ T_H_17 clones display significant similarities to mouse pathogenic T_H_17 cells in their transcriptomic patterns. The elevated expression of pro-inflammatory cytokines and chemokines in human IFN-γ-secreting T_H_17 cells and the similarity in gene expression profiles between human IFN-γ-secreting T_H_17 cells and mouse pathogenic T_H_17 cells indicates a higher pro-inflammatory capacity of human T_H_1/17 cells and additionally provides transcriptional evidence to support the role of human T_H_1/17 in the pathogenesis of human autoimmune diseases. The differential molecular signature of human IFN-γ-secreting T_H_17 cells that we identified provides a new tool that can be utilized to assess T_H_17 cells both under physiologic conditions and in association with disease.

## Methods

### Reagents

EasySep human CD4^+^ T-cell enrichment kit (catalog number 19052) for CD4^+^ T-cell isolation was purchased from StemCell Technologies. FITC-conjugated anti-human IFN-γ (clone, B27; 1:100), Alexa 647-conjugated anti-human IL-17A (clone, N49-653; 1:20), PE-conjugated anti-human IL-10 (clone, JES3-19F; 1:660), and their corresponding isotype control antibodies for intracellular cytokine staining assay were purchased from BD Biosciences. IFN-γ cytokine secretion detection kit (APC) (catalog number 130-090-762) and IL-17 cytokine secretion detection kit (PE) (catalog number 130-094-537) were purchased from Miltenyi Biotec. nCounter CodeSet HuT_H_17 was custom designed and manufactured by nanoString Technologies. Fluorescence-conjugated antibodies for cell surface staining for flow cytometry were purchased from Biolegend. RNAqeous micro total RNA isolation kit (catalog number AM1931), SuperScript VILO master mix (catalog number 11755050), TaqMan preAmp master mix (catalog number 4391128), TaqMan fast universal PCR master mix (2x) (catalog number 4352042), and qPCR primers (Supplementary Data 8) were purchased from ThermoFisher Scientific.

### Human subjects

Blood samples for T_H_17 cloning from healthy donors were obtained from the Swiss Blood Donation Center of Basel and Lugano. Informed, written consent was obtained from all donors. All uses of human material were approved by the Federal Office of Public Health (authorization no. A000197/2 to F.S.). Blood samples from MS patients and healthy controls were obtained from the Partners MS Center at Brigham and Women’s Hospital under IRB Protocol 2001P001431 and 2014P000124. Informed, written consent was obtained from all donors. MS patients were untreated for a minimum of 6 months before sampling. Disease activity was identified as a gadolinium-enhancing lesion on MRI or disease relapse within 6 months of sampling. Age- and sex-matched healthy donors did not have history of autoimmune diseases or malignancies and no acute or chronic infections. The samples from healthy donors for nCounter gene expression analysis were fresh blood samples. The MS samples and age- and sex-matched healthy control samples were frozen peripheral blood mononuclear cells (PBMCs).

### Intracellular cytokine staining

For intracellular cytokine staining for PBMC or CD4^+^ T cells, assays were carried out with staining buffers and antibodies from BD Biosciences. Briefly, cells were seeded into a 96-well plate (up to 1 × 10^6^ cell per well) and stimulated with PMA (100 ng/ml) and ionomycin (1 μg/ml) in the presence of GolgiStop for 4 h. After stimulation, cells were fixed with BD Cytofix fixation buffer and washed with BD Perm/Wash buffer. Cells in each well were equally divided into two wells, with one for intracellular cytokine staining and the other for isotype control staining. The following fluorophore-conjugated antibodies from BD Biosciences were used for staining analysis or as isotype controls: anti-CD4-pacific blue (clone: RPA-T4; 1:330), anti-IL-17A-Alexa647 (clone: N49-653; 1:20), anti-IFN-γ-FITC (clone: B27: 1:100), anti-IL-10-PE (clone: JES3-19F: 1:660), mouse IgG1-Alexa647 (clone: MOPC-21; 1:40), mouse IgG1-FITC (clone: MOPC-21; 1:100), and rat IgG2a-PE (clone: R35-95: 1:660). Stained cells were analyzed with a BD LSR II cytometer. Cytokine secretion in CD4^+^ lymphocytes was accessed with FlowJo.

### Isolation of viable T_H_ subsets from human PBMC

PBMC isolated with Ficoll-Pague PLUS (GE Healthcare) gradient centrifugation from the peripheral blood of healthy donors. Total CD4^+^ T cells were purified with the EasySep human CD4^+^ T-cell enrichment kit (StemCell Technologies). CD4^+^ T cells seeded in a 96-well plate (1 × 10^6^ cells/well) were stimulated with PMA (30 ng/ml) and ionomycin (1 μg/ml) for 3–4 h (3 h for fresh blood samples and 4 h for frozen PBMC samples). Viable T_H_1/17, T_H_17, T_H_1, and DN cells were sorted with a FACSAria (BD Biosciences) after being stained with IFN-γ and IL-17 cytokine secretion detection kits (Miltenyi Biotec) and fluorescence-conjugated anti-CD3 and anti-CD4 antibodies following the manufacturer’s suggested protocol.

### Isolation of human IL-10^−^ and IL-10^+^ T_H_17 clones

PBMC were isolated by Ficoll-Paque PLUS (GE Healthcare) separation. CD4^+^ T cells were isolated from PBMC by positive selection using CD4 magnetic microbeads (Miltenyi Biotec). CCR6^+^CCR4^+^CXCR3^–^CD45RA^–^CD25^–^CD8^–^CD14^–^CD19^–^CD56^–^ (enriched in T_H_17 cells) memory CD4 T cells were sorted with a FACSAria (BD Biosciences) and seeded at 0.6 cells per well in a 384 well plate. The following antibodies were used for FACS-based sorting: anti-CD45RA-Qdot655 (Life Technologies; clone: MEM-56; 1:1000); anti-CCR7-BV421 (Biolegend; clone: G043H7; 1:80); anti-CCR6-PE (BD Biosciences; clone: 11A9; 1:80) or anti-CCR6-BV605 (Biolegend; clone: G034E3; 1:60); anti-CCR4-PECy7 (BD Biosciences; clone: 1G1; 1:100); anti-CXCR3-PE-Cy5 or anti-CXCR3-APC (BD Biosciences; clone: 1C6; 1:20); anti-CD8-FITC or anti-CD8-PE-Cy5 (Beckman Coulter; clone: B9.11; 1:25); anti-CD25-FITC or anti-CD25-PE-Cy5 (Beckman Coulter; clone: B1.49.9; 1:25); anti-CD14-FITC or anti-CD14-PE-Cy5 (Beckman Coulter; clone: RMO52; 1:25); anti-CD19-FITC (BD Biosciences, clone: HIB19), or anti-CD19-PE-Cy5 (Beckman Coulter; clone: J3-119; 1:25); CD56-PE-Cy5 (Beckman Coulter; clone: N901; 1:25). CD4 T-cell clones were established in the presence of irradiated (45 Gy) allogeneic PBMC (25,000 cells per well) and phytohaemagglutinin (PHA-L) (1 μg/ml) in medium supplemented with IL-2 (500 U/ml). A small portion of cells of each CD4^+^ T-cell clone was used for intracellular cytokine staining assay for IL-17A (anti-IL-17A-eF660; eBioscience, catalog number 50-7179-42 clone: eBio64DEC17; 1:200), IL-10 (anti-IL-10-PE; BD Biosciences, catalog number 559330; clone: JES3-19F1; 1:20), and IFN-γ (anti-IFN-γ-FITC; BD Biosciences, catalog number 554700; clone: B27; 1:800) production. IL-10^–^ and IL-10^+^ T_H_17 clones were selected for further analysis.

### Regular quantitative real-time PCR

To measure gene expression in T_H_17 clones with qPCR, IL-10^–^ and IL-10^+^ T_H_17 clones from an individual donor were pooled. They were stimulated or not with plate-bound anti-CD3 (clone TR66, 5 μg/ml) and anti-CD28 (clone CD28.2, 1 μg/ml) (αCD3/CD28) for 4 h, or stimulated with αCD3/CD28 for 5 days followed with or without a second αCD3/CD28 treatment for 4 h. Pooled T_H_17 clone cells were subjected to RNA isolation. Total RNA was extracted with the E.Z.N.A. Total RNA kit I (Omega Bio-tek, product number R6834). qPCR analysis was run and analyzed with the ViiA 7 Real-Time PCR System (Life Technologies). Quantitative comparison between IL-10^–^ and IL-10^+^ T_H_17 clones was calculated using comparative Δ*C*_T_. Gene expression was normalized to the expression of *β2*m.

### Low-input quantitative real-time PCR

To measure gene expression in T_H_1/17, T_H_17, T_H_1, and DN cells sorted from frozen PBMC with qPCR, total RNA was isolated and digested with DNase I with the RNAqeous micro total RNA isolation kit. cDNA was synthesized with the SuperScript VILO master mix and pre-amplified for 14 cycles with the TaqMan preAmp master mix following the manufacture’s instruction. qPCR analysis was run and analyzed with the ViiA 7 Real-Time PCR System (Life Technologies) using the TaqMan fast universal PCR master mix (2x) and qPCR primers purchased from ThermoFisher Scientific. The comparative threshold cycle method and an internal control (*β2m*) were used for normalization of the target genes. Relative expression was calculated as: Δ*C*_T_ = *C*_Tgene of interest_ – *C*_Tβ2m_; ΔΔ*C*_T_ = Δ*C*_T cell subset of interest_ – mean of Δ*C*_T DN of healthy control_; the relative change of gene expression between the expression level of sample of interest and the mean expression level of all DN samples in healthy controls was given by this formula: (2 ^–ΔΔ*C*T^) × 10. All qPCR reactions were performed in duplicate.

### nCounter analysis of mRNA expression

We designed a nanoString CodeSet HuT_H_17 that constitutes a 418-gene expression detection panel specific for human T-cell activation and differentiation (Supplementary Data 1). Cell lysates were prepared from sorted T_H_1/17, T_H_17, T_H_1, and DN cells with the RLT-Plus buffer of the RNeasy Plus Mini Kit (Qiagen, catalog number 74134) and gene expression levels were generated using the CodeSet HuT_H_17 according to the protocol provided by the manufacturer (NanoString Technologies). For gene expression in IL-10^−^ and IL-10^+^ T_H_17 clones, total RNA was used for nCounter analysis following the manufacturer’s suggested protocol (NanoString Technologies).

### Data analysis

nCounter gene expression data were normalized for code count using the geometric mean, for background using the mean, and for sample content using the geometric mean of housekeeping genes (isolated human CD4^+^ T-cell subsets: *B2M*, *RPL3*, and beta actin; human T_H_17 clones: *B2M*, *GAPDH*, and beta actin) with the R 3.2.0. NanoStringNorm package. Mouse gene expression data were downloaded from GEO (GSE39820) and normalized using RMA^62^ and ComBat^63^ in GenePattern (http://www.broadinstitute.org/cancer/software/genepattern/) as previously published^12^. Genes for which multiple probes were measured on the mouse microarray were collapsed to unique genes by selecting the probe with the highest average expression across all samples. Hierarchical clustering of the human samples and of the pairwise Pearson linear correlations were done based on the 362 genes that varied across the population in an unbiased manner (unsupervised expression difference (Δ = maximum expression value – minimum expression value)≥5, defined as the difference between maximum and minimum relative gene expression values across the population without considering the subset classes) using Pearson linear correlation and average linkage in GENE-E. PCA of the isolated human CD4^+^ T-cell subsets was done in R (prcomp) based on the same 362 genes. We selected differentially expressed genes between T_H_1/17 and T_H_17 cells using the two tailed, paired Student’s *t* test followed by supervised filtering for expression differences between mean T_H_1/17 and mean T_H_17 (Δ = MEAN_TH1/17_−MEAN_TH17_)>20 for robustness. The same approach was used to select differentially expressed genes between IL-10^−^ and IL-10^+^ T_H_17 clones. GSEA was done in GenePattern using default settings (weighted scoring scheme, Signal2Noise metric, 1000 permutations)^34,64,65^ to test the enrichment of human signatures in the mouse expression profiles. Gene set overlap statistic analysis was done using the hypergeometric test in R (phyper, with *N* equal to the number of genes measured on the NanoString CodeSet). Storey’s *q*-value is used to control the FDR. Visualization of the gene expression heatmaps was done in GENE-E [http://www.broadinstitute.org/cancer/software/GENE-E/]. *Il23r* was not included in our microarray chip for mouse T_H_17 cell analysis, but we found upregulation of *Il23r* in murine pathogenic T_H_17 cells by qPCR^12^. Thus, we included *Il23r* in the murine gene expression profiles. The IPA upstream regulator analysis was performed for the differentially expressed genes (using corresponding fold changes and *p*-values) to identify key upstream regulatory molecules. Upstream regulators with *z*-scores >2 and *z*-scores <−2 represent activator and inhibitor mechanisms, respectively. The Enrichr ChEA2016 database and analytic tools were used to predict key upstream transcription factors^36,37^.

### Statistical analysis

Statistical analysis was performed with Prism 7 (GraphPad Software), R statistical software (version 3.2.0), and Excel version 14.4.7. One-way analysis of variance with Tukey’s multiple comparison test was performed to compare the differential gene expression among T_H_ subsets within HCs or MS patients when more than two cell subsets were included, while two tailed, paired Student’s *t* test was performed when only two cell subsets were included. Welch’s *t* test (unpaired *t* test with Welch’s correction) was performed to compare the differential gene expression between healthy controls and MS patients on a specific T_H_ subset. Two sided *p*-values of <0.05 were considered statistically significant.

### Code availability

Computer code used to generate results is available from the corresponding author upon request.

### Data availability

The authors declare that the main data supporting the findings of this study are available within the article and its Supplementary Information files. Gene expression data that support the findings of this study have been deposited in the Gene Expression Omnibus with the accession code (GSE104024). (All other relevant data are available from the corresponding author upon reasonable request.

## Electronic supplementary material

Supplementary Information

Description of Additional Supplementary Files

Supplementary Data 1

Supplementary Data 2

Supplementary Data 3

Supplementary Data 4

Supplementary Data 5

Supplementary Data 6

Supplementary Data 7

Supplementary Data 8
