# Supplementary material for: Transcriptional signature of human pro-inflammatory TH17 cells identifies reduced IL10 gene expression in multiple sclerosis
Source: Nat Commun. 2017 Nov 17;8:1600. doi: 10.1038/s41467-017-01571-8 (PMC5693957; doi:10.1038/s41467-017-01571-8)
Supplement: Supplementary file 1 — Supplementary Information [file 41467_2017_1571_MOESM1_ESM.pdf]

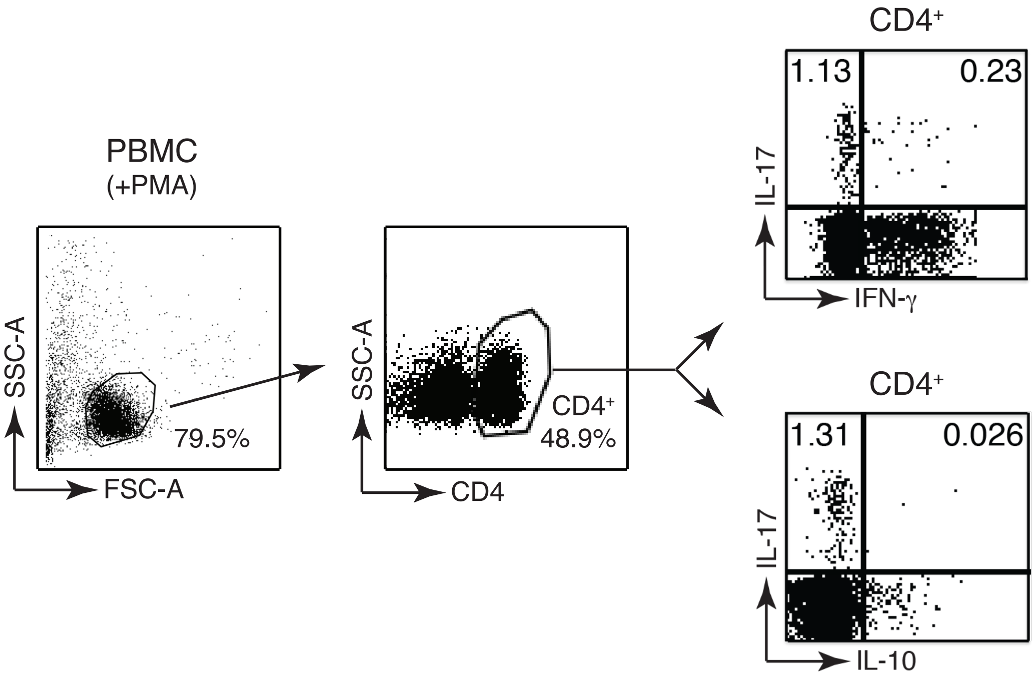

Supplementary Figure 1 Gating Strategy to determine the production of IFN-γ and IL-10 in human T_H17 cells

Isolated PBMCs were stimulated with phorbol 12-myristate 13-acetate (PMA) and ionomycin for 4 hr. Production of indicated cytokines in $CD4^+$ T cells were assessed by flow cytometry with intracellular cytokine staining assay.

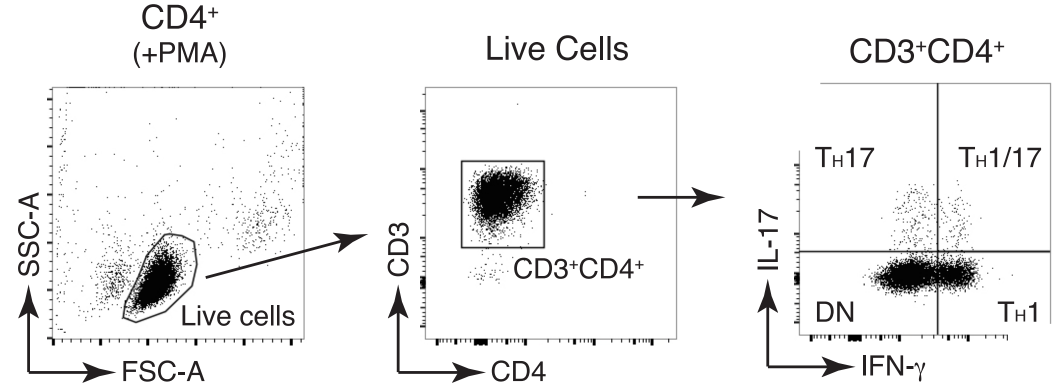

Supplementary Figure 2 Gating strategy to isolate and determine frequency of CD4$^+$ T cell subsets in total CD4$^+$ T cells in multiple sclerosis and healthy controls

CD4$^+$ T cells isolated from the peripheral blood of healthy controls (HC) and untreated patients with relapse-remitting multiple sclerosis (MS) were stimulated with PMA and ionomycin for 3-4 hr (3 hr for fresh blood samples and 4 hr for frozen PBMCs, respectively). Stimulated cells were stained with fluorescence-conjugated anti-CD3 and CD4 in combination with IFN-γ and IL-17 cytokine secretion detection kits (Miltenyi Biotec). $T_H1/17$, T_H17, T_H1 and DN cells were sorted with a FACSAria (BD Biosciences).

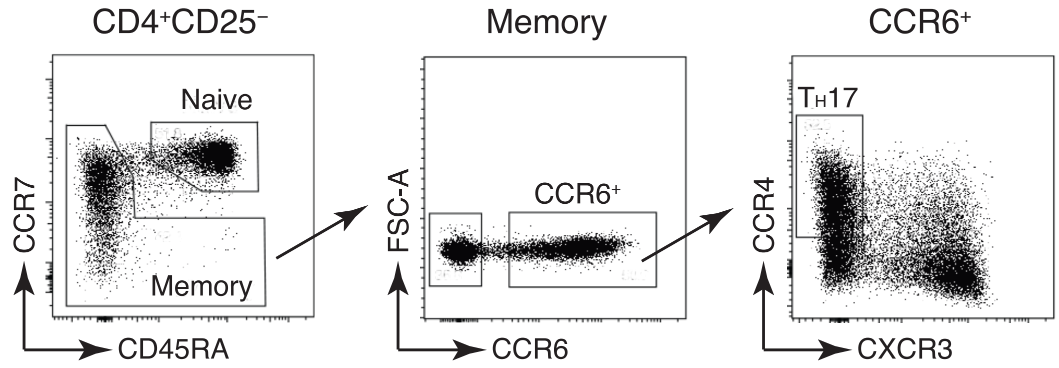

Supplementary Figure 3 Gating strategy to isolate CCR6^{+}CCR4^{+}CXCR3$^{–}$ memory CD4^{+} T cells for establishing T$_{H}$17 clones

CD4^{+} T cells isolated from PBMC were stained with anti-CD4, CD25, CCR4, CCR6, CCR7 and CXCR3 and sorted with a FACSAria (BD Biosciences) for CCR6^{+}CCR4^{+}CXCR3$^{–}$CD45RA$^{–}$CD25$^{–}$ memory CD4^{+} T cells (enriched in T$_{H}$17 cells).

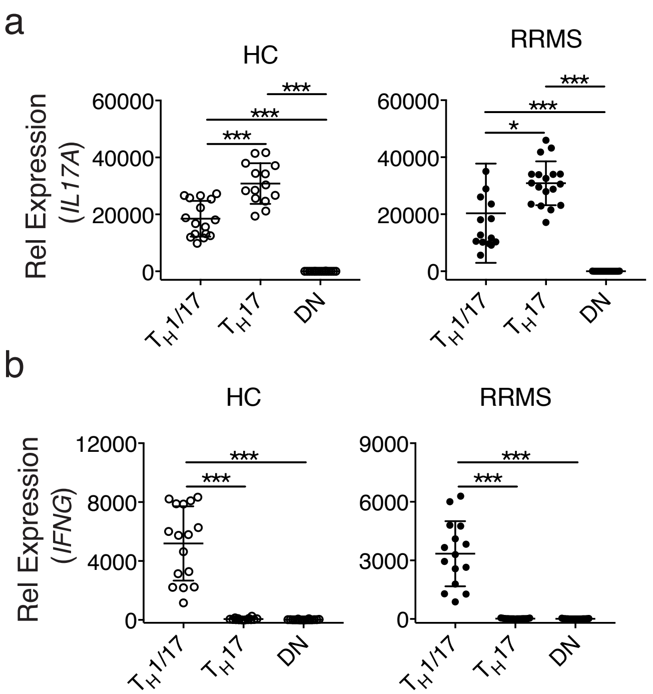

Supplementary Figure 4 *q*PCR analysis of *IL17A* and *IFNG* expression in T_H1/17, T_H17 and DN cells in MS and HC

RNA isolated from sorted T_H1/17, T_H17 and DN cells from the PBMC of untreated patients with relapse-remitting MS (n=19) and age- and sex-matched healthy controls (HC) (n=16) as described in Figure 5 was subjected to low-input *q*PCR analysis: a) *IL17A* and b) *IFNG*. *$p< 0.05$, ***$p<0.0001$, One-way ANOVA with Tukey's multiple comparison test (mean±s.d.).

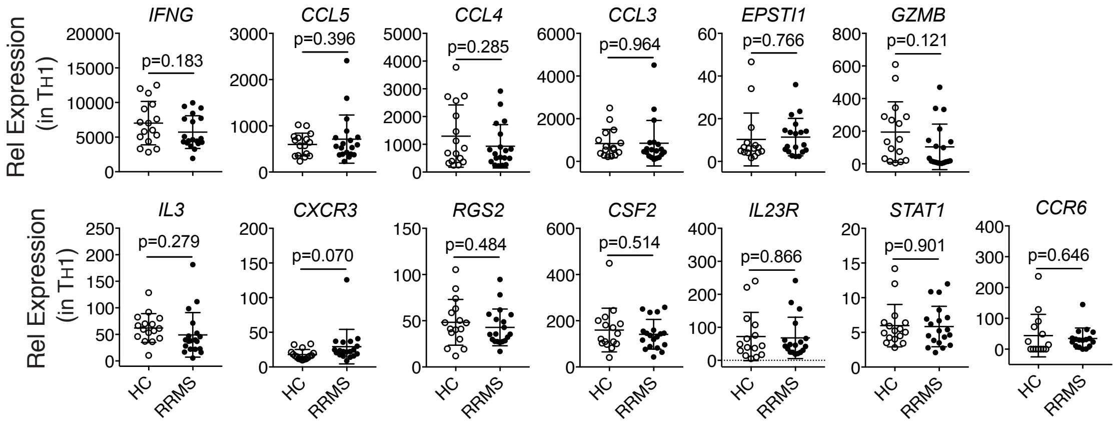

Supplementary Figure 5 Expression of the robust PreP-signature genes of human T_H1/17 cells in T_H1 cells in MS and HC

RNA isolated from sorted T_H1 cells from the PBMC of untreated patients with relapse-remitting MS (n=19) and age- and sex-matched healthy controls (HC) (n=16) as described in Figure 5 was subjected to low-input *q*PCR analysis. Welch's t test *p*-values were shown (mean±s.d.).
