# Supplementary material for: Transcriptional signature of human pro-inflammatory TH17 cells identifies reduced IL10 gene expression in multiple sclerosis
Source: Nat Commun. 2017 Nov 17;8:1600. doi: 10.1038/s41467-017-01571-8 (PMC5693957; doi:10.1038/s41467-017-01571-8)
Supplement: Supplementary file 2 — Description of Additional Supplementary Files [file 41467_2017_1571_MOESM2_ESM.pdf]

Description of Additional Supplementary Files

File Name: Supplementary Data 1
Description: **Gene list of the HuT_H17 CodeSet**

File Name: Supplementary Data 2
Description: **The presence of murine and human T_H cell signature genes in nCounter CodeSet HuT_H17**. a, murine pathogenic T_H17 signature genes in experimental autoimmune encephalomyelitis (EAE) identified by Lee et al.[12]. b, murine EAE signature genes enriched in human myelin-specific $CCR6^+$ $CD4^+$ T cell libraries derived from patients with MS[19]. c, EAE signature genes enriched human $CD45RO^+CCR7^{lo}CCR6^+CCR4^{lo}CXCR3^{hi}$ memory effector T_H17.1 cells from healthy donors[33]. d, EAE signature genes in nCounter CodeSet HuT_H17. n.a., not available or not presented; +, gene expressed up-regulated; -, gene expression not up-regulated.

File Name: Supplementary Data 3
Description: **Up and down-regulated genes in T_H1/17 cells compared to T_H17 cells**. Differentially expressed genes between T_H1/17 and T_H17 cells were select with the two-tailed, paired Student's t-test $p<0.05$ followed by supervised filtering for expression differences between Mean T_H1/17 and Mean T_H17 ($\Delta = MEAN_{TH1/17} - MEAN_{TH17}$) >20 for robustness. Abbreviations: Mean T_H1/17, mean gene expression value of T_H1/17 cells; Mean T_H17, mean gene expression value of T_H17 cells.

File Name: Supplementary Data 4
Description: **Genes differentially expressed between human *ex vivo* T_H1/17 vs. T_H17 cells but not enriched in human and mouse pathogenic/non-pathogenic T_H17 gene set enrichment analysis comparisons**. Differentially expressed genes between T_H1/17 and T_H17 cells were select with the two tailed, paired Student's t-test $p<0.05$ followed by supervised filtering for expression differences between Mean T_H1/17 and Mean T_H17 ($\Delta = MEAN_{TH1/17} - MEAN_{TH17}$) >20 for robustness. Abbreviations: Mean T_H1/17, mean gene expression value of T_H1/17 cells; Mean T_H17, mean gene expression value of T_H17 cells.

File Name: Supplementary Data 5
Description: **Up and down-regulated genes in IL-10^- T_H17 clones compared to IL-10^+ T_H17 clones**. Differentially expressed genes between IL-10^- and IL-10^+ T_H17 clones were select with two tailed, paired Student's t-test $p<0.05$ followed by supervised filtering for expression differences between Mean IL-10^- T_H17 clone and Mean IL-10^+ T_H17 clone ($\Delta = MEAN_{IL\text{-}10-\ TH17} - MEAN_{IL\text{-}10+\ TH17}$) >20 for robustness. Abbreviations: Mean IL-10^-, mean gene expression value of IL-10^- T_H17 clones; Mean IL-10^+, mean gene expression value of IL-10^+ T_H17 clones.

File Name: Supplementary Data 6
Description: **The predicted upstream transcription factors for T_H1/17 differentiation identified with Enrichr ChEA2016 analysis**
All transcription factors with Benjamini-Hochberg adjusted *p* value smaller than 0.05 are shown.

File Name: Supplementary Data 7
Description: **The predicted upstream transcription factors for IL-10^{-} clone differentiation identified with Enrichr ChEA2016 analysis.** All transcription factors with Benjamini-Hochberg adjusted *p* value smaller than 0.05 are shown.

File Name: Supplementary Data 8
Description: **List of *q*PCR primers**
